# Evaluating the Effect of an Experimental Sesame Gel on Human Enamel: Atomic Force Microscopy Study

**DOI:** 10.30476/DENTJODS.2021.87878.1293

**Published:** 2022-06

**Authors:** Faeze Hamze, Leila Ghasemi, Mohammad Kamalinejad

**Affiliations:** 1 Dept. of Operative, Shahed University of Tehran, Tehran, Iran; 2 Dept. of Operative, Dental School, Ilam University of Medical Sciences, Ilam, Iran; 3 School of Pharmacy, Shahid Beheshti University of Medical Sciences (SBMU) Tehran, Iran

**Keywords:** Herbal, Sesame, Fluoride, Remineralization, Enamel, Surface Roughness

## Abstract

**Statement of the Problem:**

Every effort for increasing the calcium concentration in the saliva would be beneficial for prevention of dental caries. Regarding this issue, the natural products could be considered safer and more cost effective. Sesame is rich in calcium but the data about the effect of sesame on enamel roughness is inadequate.

**Purpose:**

This study aimed to assess the effect of an experimental sesame gel on the surface roughness of human enamel by using atomic force microscopy (AFM).

**Materials and Method:**

In the current experimental study, fifteen enamel slices with 1mm thickness were prepared. They were polished and acid etched to produce a substantial rough surface prior to the first AFM analysis. The enamel blocks were randomly divided into three groups treated with distilled water, fluoride gel, and an experimental prepared sesame gel correspondingly. The treating agent was applied for 3minutes at 0, 8, 24, and 48h intervals and washed by distilled water after each cycles. Ultimately, the final AFM micrographs were prepared. The statistical analysis was performed using paired t-test, one-way ANOVA, and Tukey Post Hoc tests (α=0.05).

**Results:**

Statistical analysis revealed that the surface roughness was significantly reduced in both sesame and fluoride groups (*p*= 0.017 and 0.018, respectively) while the control group (distilled water) were not noticeably changed (*p*= 0.12). The control group had statistically significant difference with both the sesame and the fluoride groups (*p*= 0.007 and 0.007, respectively) while the there was no significance difference between sesame and fluoride groups (*p*= 0.997).

**Conclusion:**

Following demineralization by acid etched process, the sesame gel significantly reduced surface roughness of enamel and its effect was similar to fluoride gel.

## Introduction

Dental caries is a major public health problem and the most common infectious disease among human population [ 1 ]. Since most dental treatments are painful and costly, patients have become more interested in preventing tooth decay rather than seeking treatment [ 2 ].

Carious lesions are the result of demineralization of the tooth microstructure due to the acid produced by cariogenic bacteria, which subsequently lead to cavity production in tooth structure [ 3 ]. Therefore, remineralizing agents that expose the tooth surface to mineral-rich saliva [ 4 ] might slow down or even prevent the decay process [ 3 ]. Despite the rich history of the benefits of fluoride in tooth care and its robust status as the standard low-cost therapeutic agent in dental clinics [ 2 ], it has some shortcomings such as toxicity [ 5 - 8 ]. Consequently, recent studies are conducted to offer some innovative and very effective preventive agents such as bioactive glasses, amorphous calcium phosphate, and so on. Although these agents showed remarkable remineralization potentials, their high cost and the need for modern mobile technologies have limited their use in dental clinics [ 3 , 6 , 8 - 10 ]. 

In recent years, dental researchers have been encouraged to investigate the effects of natural products on dental caries [ 11 ]; these products generally show minimal or no side effects and they are economic [ 12 ]. The research shows that some foods contain anti-decay agents such as calcium, phosphate, and casein. Hence, these foods might potentially be able to contribute to remineralization process and reduce the occurrence of caries [ 13 ]. Moreover, green leafy vegetables, seeds, and legumes are rich sources of calcium [ 14 ], among which, sesame is most remarkable and famous one [ 15 ].

Besides that, in traditional medicine, oil therapy has been widely recommended to strengthen the teeth, jaws, gums, and prevent teeth decay [ 16 ]. Sesame oil has been documented as one of the most frequent prescribed oils in traditional medicine for this purpose [ 16 ]. Previous studies have provided evidence that oil therapy with sesame oil could be used for prevention of tooth decay [ 17 - 19 ]. It is also reported that the sesame oil could noticeably reduce the plaque formation and consequently inhibit the plaque induced gingivitis [ 4 , 18 ]. In addition, the antibacterial potential of sesame oil has been broadly documented against the cariogenic bacteria particularly *Streptococcus mutans*(S. mutans) [ 19 - 20 ]. Therefore, the sesame seed could be considered as an effective substance in preventive dentistry [ 4 , 18 - 20 ]. Moreover, numerous anti-caries agents have remineralization capability regarding their calcium or other mineral contents. Current studies have shown that sesame is one of the richest herbal sources of calcium [ 14 , 17 ].

However, the available data regarding the effect of sesame on surface enamel is quite sparse. In an *in vitro* study, it has been shown that this plant could increase the microhardness of human enamel though the effect of sesame to reduce the surface roughness especially after acid incorporation has not been investigated [ 21 ]. The effect of sesame on remineralization process is still disputed. Therefore, this study aimed to investigate the effect of an experimental sesame gel on the surface roughness of human enamel by employing atomic force microscopy (AFM).

## Materials and Method

### Preparation of sesame extract gel

In order to prepare the extract, 200 gr of black sesame seed, purchased locally, were immersed in boiled water for 48 h. Consequently, they were filtrated, the solvent was evaporated, and the residues were weighed that was roughly 20gr. Finally, it was admixed with 80 gr of Carbomer 934 to produce an applicable 20% gel.

### Preparation of the teeth

The Research Ethics Committee of Kerman University of Medical Science (2/2017) approved the study. Eight healthy human premolars, extracted for orthodontic reasons, were selected for this study. The teeth were stored in tap water while the water was replaced once a week. At the beginning of the study, the teeth were disinfected by immersion in 5.25% sodium hypochlorite solution for one hour, and then stored in distilled water.

In order to discard the unacceptable samples, the teeth were cleaned with a low-speed handpiece using brushes and slurry of pumice. The teeth were examined by naked eye and dental loupes (×2.5) and the cracked teeth were disregarded from the study. Moreover, considering WHO guidelines, the study samples were confirmed to be caries free [ 21 ]. 

The selected teeth were mounted in clear polyester resin. Subsequently, the outer most of both buccal and lingual enamel (with 1mm thickness) was sectioned using CNC cutting machine (Nemo Co. Mashhad Iran). Thereafter, the inner surface of the sliced enamel was serially wet polished by all four color set of polishing silicon carbide paper discs (Sof-Lex™, 3M, USA) while each disk was applied for 30 seconds and discarded after polishing three samples. Finally, the specimens were washed in water with an ultrasonic cleaner (Branson Ultrasonics Corp, Danbury, CT, USA) for 2 minutes. Ultimately, fifteen polished enamel slices were selected for the rest of the study.

### Demineralization

In order to induce an extremely rough surface, the polished enamels were etched with 35% phosphoric acid (Ultra-Etch, Ultradent products, USA) for 20 seconds and then washed for another 20 seconds with water spray.

### Atomic force microscopy (AFM)

After erosive process, the surface topography AFM images of the sample were analyzed by Scanning Probe Microscopy (SPM) model DME (Doalscope^TM^ DS95). Briefly, three regions (5m5m) were randomly chosen on each flat enamel surface.

### Remineralizing treatment

The fifteen samples were randomly divided into three groups (n=5) and were exposed to the following agents: (1) the experimental sesame gel, (2) 2% sodium fluoride gel (Masterdent, USA), which was served as positive control, and (3) distilled water (as negative control).

The samples in each group received 0.5cc of either treatment agents for 3minutes at 0, 8, 24, and 48h and washed by distilled water after each cycle. The specimens were stored in distilled water between these intervals [ 22 ].

The samples were then washed thoroughly by distilled water for 3 minutes to ensure the remnants of the agents were removed. Finally, the surface roughness of each enamel slice was measured again in three regions by the AFM.

### Statistical analysis

The roughness values measured before and after remineralizing treatments in each group were compared, using paired t-test. The values of the three groups, yielded before or after treatment, were compared with each other by employing one-way ANOVA followed by Tukey post Hoc tests (α=0.05).

## Results

**Figure 1:**
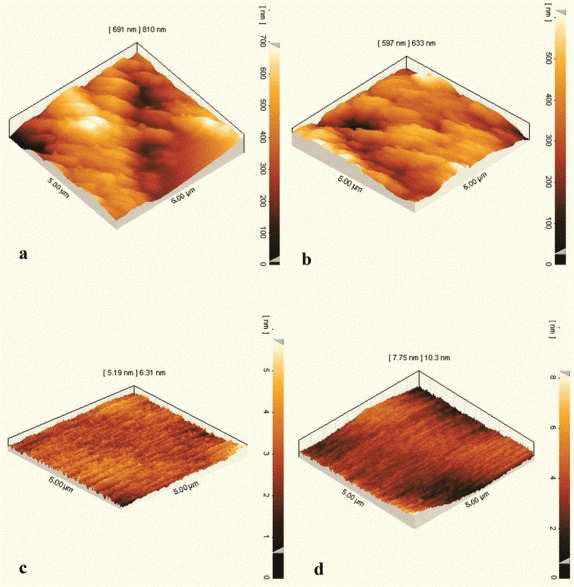
Atomic force microscopy micrographs representing apparent more roughness in acid etched (a) and water immersed groups (b); while the remineralized groups treated by either sesame (c) or fluoride (d) shows significantly smoother surfaces

The sample AFM micrographs are demonstrated in Figure 1. The figure shows that the surface irregularities were much more visible in control group and more pronounced after incorporation of acid etching and before remineralization treatment.

**Figure 2:**
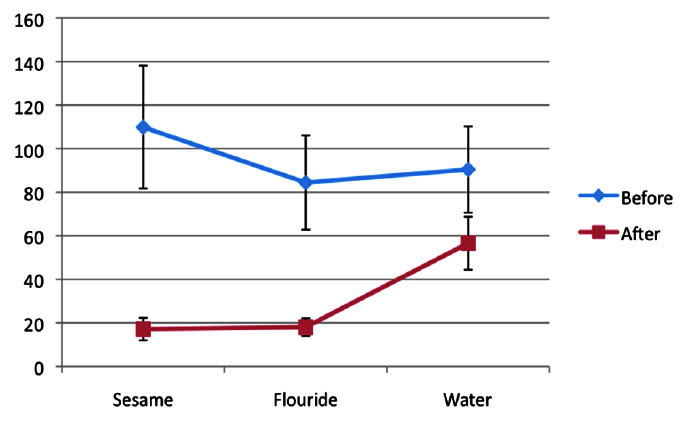
The mean S.D of surface roughness related to three groups before and after exposing to Sesame (1), Fluoride (2), and Water (3)

Moreover, the mean surface roughness values S.D of all sub-groups are depicted in Figure 2. As it can be seen, after acid etching and before remineralization, there were no statistically significant difference among the three groups (*p*= 0.782) while after treatment the difference was significant (*p*=0.03). Moreover, the Tukey pairwise comparison revealed that there was no significance difference among fluoride and sesame treated groups (*p*= 0.997); but the control group (distilled water) had significant difference with both of the remineralized groups (*p*= 0.007 and 0.007 for fluoride and sesame respectively) ( Table 1).

**Table 1:** The *p* values related 1 to Tukey pairwise comparison of the subgroups at the base line (above the diagonal) and after applying the treatment agent (below the diagonal)

	Sesame	Fluoride	Water
Sesame	-	0.772	0.829
Fluoride	0.997	-	0.982
Water	0.007	0.007	-

## Discussion

Our results showed that applying either fluoride gel or the experimental sesame gel significantly reduced the surface roughness of enamel comparing to the control group.

This is an interesting observation because it revealed that the experimental sesame gel was as effective as fluoride gel and could compensate the surface irregularities induced by acid etching of enamel.

This finding is in accordance with our previous investigation, which reported enhanced surface micro-hardness of enamel exposed to a sesame-extracted gel [ 21 ]. Since the process of preparing the sesame gel was completely similar in both studies, it could be inferred that the relative smoother surface of the enamel in sesame group in the current study might be the result of elevated superficial mineral content. In this regard, we showed that the application of sesame gel on the tooth surface increases the microhardness of the enamel, but we found no significant difference between the sesame and fluoride groups [ 21 ]. In that study, sesame extract was in gel form to facilitate its placement on the tooth surface. However, we did not investigate the remineralizing effect of sesame nor its impact on surface irregularities. Since reducing the surface irregularities might diminish the plaque accumulation on the surface enamel, it may prevent caries initiation. 

Sesame seeds are rich in oil (50%) and protein (20%) [ 23 ]. The study of Kamchan *et al*. [ 14 ] reported that sesame seed has a high level of calcium but has limited bioavailability because of the presence of oxalate, phytate and fiber [ 14 ]. 

Poneros‐Schneier *et al*. [ 24 ] investigated the bioavailability of calcium in several foods were investigated, and reported that sesame oil had a calcium bioavailability of about 65% [ 24 ]. In addition, some studies claimed about the drastic benefits of sesame for inhibition of plaque formation in clinical situations. Saravanan *et al*. [ 18 ] showed the effect of sesame oil on plaque-induced gingivitis, and found that sesame oil could reduce plaque development, gingivitis, and microbial count in oral cavity [ 18 ]. This effect can be due to not only the antimicrobial activity of sesame oil but also the cleansing effect of mouthwash. 

Sesame is considered as a natural gift of calcium source [ 21 ]. Our study showed that for remineralization of etched enamel, the sesame extract was as effective as fluoride. This could be considered as an imperative finding because fluoride varnish is a synthetic product and its remineralizing capacity has been broadly proven. Our natural sesame extract was statistically comparable to this kind of agent, which could be considered as a quite beneficial outcome in preventive dentistry. In other words, it seems that the sesame could be potentially considered as a natural gift for remineralization of the human tooth. 

In this research, we turned the sesame extract into a gel to facilitate its placement on the tooth surface. However, the ease of access to that gel is debatable. In other words, if the evidence establishes that sesame oil has the same properties as its gel, it will be more reasonable to recommend sesame oil, since it is more easily available on the market.

The remineralization properties of substances can be studied through several methods, including microhardness test and chemical analysis of tooth surface to measure phosphorus and calcium ions. However, since mineral deposition (required for remineralization) decreases the surface roughness of the tooth, any test that measures the surface roughness of the enamel can probably yield valuable information in this regard. Among the various methods of surface roughness analysis, the AFM is one of the most widely accepted method in dental researches; it has been frequently reported as a convenient test [ 22 , 25 ].This technique provides high-resolution 3D images of the studied surfaces [ 18 ], thus allowing grooves and features to be measured in nanometer scale [ 25 ]. In addition, it has been shown that this method would not cause the dehydration of enamel surface; it could be considered as a good microscopic method for the analysis of biological samples under normal conditions [ 22 ]. Some studies have employed this method to evaluate the surface roughness of the enamel and evaluated the demineralizing effect of acidic agents or compensating effect of various protective and remineralizing agents [ 22 , 25 ]. 

In our study, the AFM micrograph showed the protective effect of sesame gel comparable to the fluoride gel. Since both types of these gels are water-soluble and the specimens were thoroughly washed with distilled water prior to AFM analysis, the reduced surface irregularities could not be attributed to the possible remained debris of the gels. Therefore, the observed surface smoothening would be the result of chemical deposition. Consequently, the sesame gel could be considered as re-hardening agent for enamel surface, confirmed by the microhardness test in previous research [ 21 ]. Nevertheless, further investigations using other tools such as SEM/EDAX is strongly suggested in future studies.

It should be mentioned that the in vivo situation is somehow different from the natural clinical situation since the saliva as well as the acquired pellicle would alter the impact of this gel [ 22 ]. Accordingly, future clinical surveys are needed to evaluate the clinical effect of sesame on tooth remineralization.

## Conclusion

The results of the current research revealed that both the fluoride gel and the experimental sesame gel had noticeable beneficial effect for compensating the surface roughness of human enamel. Therefore, the sesame extract could be considered as a potential agent in preventive dentistry while further clinical researches are strongly suggested.

##  Conflict of Interest:

The authors declare that they have no conflict of interest.
